# Do Long-Term Natural Disasters Influence Social Trust? Empirical Evidence from China

**DOI:** 10.3390/ijerph18147280

**Published:** 2021-07-07

**Authors:** Yao Li, Haoyang Li, Jianqing Ruan

**Affiliations:** 1 School of Economics, Zhejiang University of Finance and Economics, Hangzhou 310018, China; lisantu@zufe.edu.cn; 2 Institute for Advanced Research, Shanghai University of Finance and Economics, Shanghai 200433, China; lihaoyang@mail.shufe.edu.cn; 3 China Academy for Rural Development, Zhejiang University, Hangzhou 310058, China

**Keywords:** long-term natural disasters, social trust, China

## Abstract

The natural environment is one of the most critical factors that profoundly influences human races. Natural disasters may have enormous effects on individual psychological characteristics. Using China’s long-term historical natural disaster dataset from 1470 to 2000 and data from a household survey in 2012, we explore whether long-term natural disasters affect social trust. We find that there is a statistically significant positive relationship between long-term natural disaster frequency and social trust. We further examine the impact of long-term natural disaster frequency on social trust in specific groups of people. Social trust in neighbors and doctors is stronger where long-term natural disasters are more frequent. Our results are robust after we considering the geographical difference. The effect of long-term natural disasters remains positively significant after we divide the samples based on geographical location. Interestingly, the impact of long-term flood frequency is only significant in the South and the impact of long-term drought frequency is only significant in the North.

## 1. Introduction

Trust plays an essential role in explaining regional economic growth differences. Economic transaction depends on trust, as risk preference and trust behavior are important determinants of most economic decisions [1]. Trust also affects schooling and the rule of law directly, which raises economic growth rates [2]. The effects of trust on institutional development, corruption, subjective life satisfaction, willingness to pay, earnings management and corporate managers’ socially responsible activities have also been examined in various ways [3,4,5,6,7,8,9,10,11,12].

However, which factors influence social trust? How is social trust formed? A growing body of literature on social trust has emphasized the significant impact of national culture and household background on regional social trust [7,13,14,15,16,17]. La Porta, et al. [11] find a negative association between trust and the dominance of a strong hierarchical religion in a country. Economic system is important and economic freedom may enhance social trust [14]. Knack and Keefer [13] suggest that trust is usually stronger in nations with higher and more equal incomes, and with better-educated and ethnically homogeneous populations and institutions that restrain the predatory actions of chief executives. Their opinions are supported by Bjørnskov [7], who indicates that income inequality and ethnic diversity reduce trust. Dinesen, et al. [17] reviewed the existing literature on the relationship between ethnic diversity and social trust, and found a statistically significant negative relationship between ethnic diversity and social trust across all their studies. Family cultural background can also affect social trust [15,18]. Drawing upon a nationally representative sample of the German population, Gereke, et al. [18] found that household poverty partially accounted for lower levels of trust. Moreover, with the data from a random web survey of college students, Valenzuela, et al. [19] investigated the impact of online social networks on social trust and found a positive association between online social networks and social trust. Recent study also provides positive evidence for the relationship between trust and genetic factor [20].

As one of the most essential factors in human history, the natural environment plays a vital role in people’s psychological performance. The relationships have been examined in various ways [21,22,23]. Chew, et al. [21] study the causal effect of haze on human personality preferences through natural experiments and find that people are more risk-averse, more impatient and more selfish in haze weather. Hanaoka, et al. [22] indicate that earthquakes will change people’s risk appetite and they show that men who live in high-frequency earthquake areas will be more adventurous and radical, while women will be more risk-averse. Bernile, et al. [23] find that natural disasters experienced in childhood play an important role in risk preference. People will be more risk-averse and conservative if they experience severe natural disasters.

Despite the massive physical and economic damage, natural disasters also affect social trust as societies need to work together to meet naturally occurring events [24]. Many scholars have verified the relationship between natural disasters, such as earthquakes and tsunamis, and social trust [25,26,27,28]. Veszteg, et al. [29] found that mutual trust increased following the massive Tohoku earthquake that hit Japan in spring 2011. The influence of natural disasters on social trust may last for a long time. Lee [30] suggests that the disaster experience is positively associated with trust: Japanese citizens with disaster experience had higher levels of in-group and out-group trust than those without disaster experience, and Tohoku residents showed higher levels of out-group, generalized, and political trust than the residents of other regions. Natural disasters affect not only social trust among people but also affect public trust in organizations. Nakayachi [27] conducted two surveys to measure the public’s trust in risk-managing organizations, before and after the Tohoku Earthquake and the results showed that trust decreased in risk-managing organizations that deal with earthquakes and nuclear accidents, whereas trust levels related to many other hazards, especially in areas not touched by the Tohoku Earthquake, remained steady or even increased. Furthermore, scholars also investigate the reasons why public trust and political trust may change after natural disasters. The pre-disaster distrust, local officials’ impolite manners, and the gap between public expectations and the local government capacity in disaster relief impair trust in the local government [26]. You, et al. [28] used the Wenchuan earthquake as a natural experiment and found that public trust changes during the whole process of natural disaster. Due to the extensive media coverage, public trust in government officials rose significantly after the earthquake. Experimental economics methods have also been employed in investigating the impact of endogenous shocks such as natural disasters on social trust [1,31]. Ahsan [1] conducted a risk and trust game in Bangladesh and found that natural disasters could significantly reduce people’s risk-taking attitudes, whereas catastrophic events had no influence on trusting behavior. Fleming, et al. [31] conducted a trust game experiment in earthquake-affected and non-affected rural villages after the 2010 Chilean earthquake, suggesting that trust levels did not differ across areas.

Even though there is a growing body of literature regarding natural disasters and its impact on social trust, most of them focus on massive natural disasters’ transitory influence. Trust is relatively stable over time [7,15,25,32]. Uslaner [15] showed that immigrants’ descendants whose grandparents came to the United States from countries that have high levels of trust tend to have higher levels of generalized trust. The natural disasters’ effect is not transitory, and it persists and actually increases over time [32]. The experiments that Cassar et al. [25] conducted in rural Thailand also supported this argument. They found that the 2004 tsunami led to substantial long-lasting increases in risk aversion, prosocial behavior, and impatience. When human beings face external risks, they need to cooperate to resist threats and social trust is improved [24,29,30]. In the study of disasters, social association is regarded as one of the most basic social units that respond to disasters [33,34]. Therefore, long-term natural disasters have played an essential role in enhancing social trust and social relationship networks in humankind’s long-term evolution [24,25]. This paper uses long-term agricultural production natural risk data and micro-survey data to verify the impact of long-term natural disasters on social trust.

China is a country with a long history of natural disasters such as floods, droughts and earthquakes. Among all kinds of disasters, floods and droughts disasters have the widest distribution and the most damage to agricultural production and the human race in China [35]. Thus, we use long-term floods and droughts data which are derived from the Atlas of Droughts and Floods Distribution in China over the Last 500 Years and its extended data to estimate the long-term natural disaster frequency. We argue that long-term natural disasters affect people’s behavior in the long-term process and strengthen the level of cooperation to resist risks, thereby promoting regional social trust. As far as we know, our paper is one of very few to explore how long-term natural disasters influence social trust.

Employing data from China Family Panel Studies (CFPS) in 2012, we use the Logit model to investigate the impact of long-term natural disaster frequency on social trust. We find that people in regions with higher long-term natural disaster frequency are more likely to trust others. With the ordered Probit model, we further address the impact of long-term natural disaster frequency on the trust of specific groups such as parents, neighbors, Americans, strangers, cadres, and doctors. The results indicate that the influence of long-term natural disasters varies from group to group. In areas with higher long-term natural disaster frequency, people are more likely to trust neighbors and doctors; however, the impacts of long-term natural disaster frequency on parents, Americans, strangers and cadres are not significant. A possible explanation is that people have to seek cooperation and help from neighbors and doctors in areas where long-term natural disasters are frequent.

We further compare the impact of long-term natural disaster frequency on social trust in southern China and northern China. The evidence indicates that natural disaster frequency significantly impacts trust in both regions. Interestingly, flood frequency has a significantly positive relationship with social trust only in southern China and the impact of long-term drought frequency is significant only among the northern China samples. This may be due to the dominance of floods in the South and droughts in the North.

The remainder of the paper proceeds as follows. In Section 2, we present data sources and descriptive evidence. Section 3 shows the empirical analysis of the impact of long-term natural disasters on social trust. In Section 4, we further discuss the results based on geographical division. The impact of long-term natural disasters on social trust is investigated respectively in southern China and northern China. Section 5 concludes the paper.

## 2. Data Sources and Descriptive Statistics

### 2.1. Data of Social Trust

The natural environment has profoundly affected and restricted human behavior and life, especially in the early years with low productivity and underdeveloped technologies. The natural environment has an irreplaceable influence on human society’s formation and it shapes human personality characteristics, such as risk appetite, cooperation preference, and dedication [36]. Following Bjørnskov [7], Nannestad [37] and Uslaner [38], we use similar questions from large-scale survey to measure social trust. The social trust data that we employ in this article is from the 2012 China Family Panel Studies (CFPS). China Family Panel Studies is a national, large-scale, multidisciplinary social tracking survey project which is conducted by China Social Science Research Center of Peking University since 2010. The samples in this survey cover 25 provinces across the country. The CFPS survey questionnaire has four questionnaires for specific groups: community questionnaire, family questionnaire, adult questionnaire and children questionnaire.

We use adult questionnaire data in 2012 in this article. The 2012 China Family Tracking Survey (CFPS) adult data observations used in this paper are distributed in 231 counties, covering 25 provinces, cities and autonomous regions across the country. In the adult questionnaire tracking survey, two questions directly measure social trust. The first question is: “Generally speaking, do you think most people can be trusted, or you can’t be too careful”? The respondents have two options: one is “most people can be trusted”, and the other one is “you can’t be too careful”. We use the answer to this question to represent the generalized trust level of the respondent. Specifically, if the respondent chooses the first option, his generalized trust level is high, and we set the variable generalized trust = 1. Otherwise, generalized trust = 0. Besides, this survey has another question regarding social trust, which is “Please score your trust for the following categories of people from 0–10: parents, neighbors, Americans, strangers, cadres, and doctors. 0 means very distrustful, and 10 means very trusting”. The second question’s answer shows respondents’ trust in some specific groups of people that they often come into contact with or barely come into contact with. By adding up the scores to those six specific groups of people, we can measure the average level of social trust in specific groups. The descriptive statistics of the general trust and the trust of specific groups of the samples are involved in Table 1. When human beings face external risks such as flood and drought, they need to cooperate to resist threats. Thus, social trust may be influenced by natural disasters.

### 2.2. Data of Long-Term Natural Disasters and Other Variables

**Figure 1:**
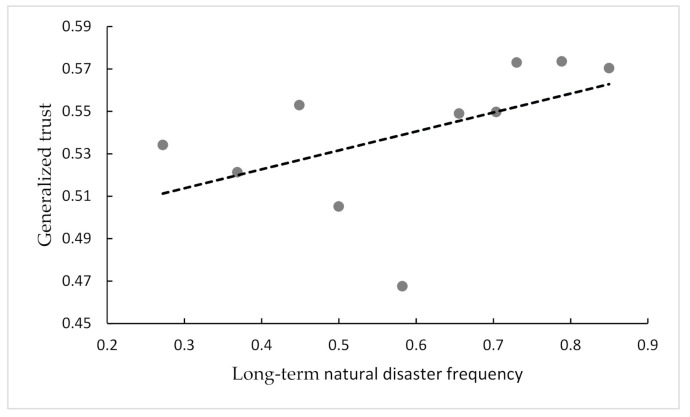
Scatter plot of generalized trust and long-term natural disaster frequency.

**Figure 2:**
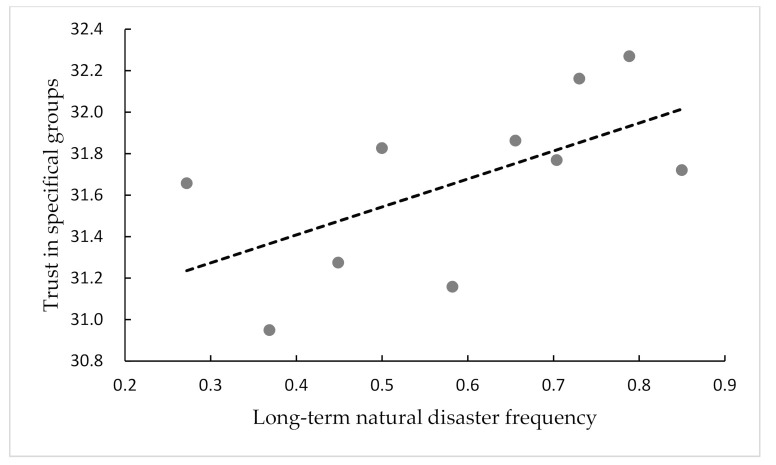
Scatter plot of trust in specific groups and long-term natural disaster frequency.

According to data availability and modeling accuracy, conventional measures of natural disaster risk are developed, namely as risk, risk grade, and risk level, for the convenience of explanation [39]. Hirabayashi, et al. [40] use different climate models to calculate global flood risk at the end of this century. Because of the limitation of historical data, we calculate long-term natural disaster frequency as the indicator of long-term natural disasters. Floods and droughts are the major natural disasters that affect agricultural production and the human race in China [35]. Thus, in this paper, we use the occurance of floods and droughts to estimate the long-term natural disaster frequency. The natural disaster data we use are derived from the Atlas of Droughts and Floods Distribution in China over the Last 500 Years and its extended data. This dataset contains the meteorological records of floods and droughts from 120 observation spots from 1470 to 2000 in China. By adding the degree of floods and droughts, the county level’s historical disaster frequency in every year is obtained. Then we can obtain the mean natural disaster frequency as the long-term natural disaster frequency for every county. Figure 1 and Figure 2 show that long-term natural disaster frequency correlates positively with generalized trust and trust in specific groups. When exogenous shocks such as long-term natural disasters in the region are more frequent, local people are more inclined to trust each other and cooperate to enhance their ability to resist risks.

To verify the relation between long-term natural disasters frequency and social trust, we also control other variables such as individual’s gender (1 = male, 0 = female), age, education, marriage, religious beliefs (1 = yes, 0 = no), hukou (1 = urban, 0 = rural), household income, ethnic minorities ratio, and per GDP (ln). Individual-level variables, such as gender, age, education, marriage, religious beliefs, and hukou, are obtained from the adult questionnaire. Household income comes from the household questionnaire. Finally, ethnic minorities ratio and per GDP data are embraced from China’s sixth census in 2010 and China Regional Economic Statistical Yearbook (2013), respectively. Descriptive statistics are detailed in Table 1.

## 3. Empirical Methodology and Results

Our research investigates whether long-term natural disaster frequency has an effect on social trust. Since the general trust level variable is binary (1 = most people can be trusted, 0 = you can’t be too careful), we use the Logit model to conduct the regression analysis, and the results are shown in Table 2. We first use long-term natural disaster frequency as the key independent variable to explain the general trust level, and the results are listed in Columns R1 and R2. Long-term natural disaster frequency has a significant positive effect on generalized trust. After controlling other variables, the influence of long-term natural disaster frequency is still significant. Flood and drought are different natural disasters and people’s cooperation behavior may vary with different kinds of natural disasters. Thus, we also testify the impact of floods frequency and droughts frequency on generalized trust. The results in Columns R3-R6 in Table 1 indicate that floods and droughts still have significant positive impacts on generalized trust. Compared with droughts, floods have a greater impact on generalized trust. The coefficient of flood frequency is larger than the coefficient of drought frequency.

We further analyze whether the effect of long-term natural disaster frequency on trust depends on the person to whom the trust is extended to the average level of social trust in specific groups as the dependent variable. Table 3 reports the results of ordinary least squares (OLS) regression. Similar to the regression results of general trust levels, the frequency of natural disasters has a significant positive impact on the average level of social trust in specific groups. The significant impact mainly comes from drought disasters instead of flood disasters, which imposes significant positive effects on the overall trust in certain groups of people.

Does the effect of long-term natural disaster frequency on trust depends on the person to whom the trust is extended to? We respectively examine the influence of long-term natural disaster frequency on the trust of six specific groups of people (parents, neighbors, Americans, strangers, cadres, and doctors). The Likert Scale form of this question in the questionnaire is essentially an orderly choice one, so we use the Ordered Probit model to conduct the analysis. The results are shown in Table 4. For different groups of people, the impact of long-term natural disaster frequency has significant differences. Long-term natural disasters significantly affect the trust of the local population in their neighbors and doctors. The higher the frequency of natural disasters, the higher the local people’s trust in neighbors and doctors. This is consistent with the hypothesis of this article: in areas where the natural risk of agricultural production is high and natural disasters are frequent, people often seek cooperation from neighbors, and when they encounter physical injuries during the disasters, they need to seek help from doctors. Therefore, the trust inward neighbors and doctors will be significantly higher. In contrast, the impact of long-term natural disasters on the trust in Americans and strangers, those that do not usually come into contact with the respondents, is both economically and statistically insignificant.

## 4. Further Discussion on Geographical Division

China has a vast territory and a large geographical span. There are big differences in geographical environment and climatic conditions between the South and the North. In addition, the different types of major food crops in the North and South will lead to differences in human behavior when resisting the risk of external natural disasters, which in turn affects the general trust in others in different regions. In order to ensure the robustness of the results of this paper, based on the previous research, this paper divides the respondents to those residents in the North and in the South. Table 5 reports the results of this split-sample regression. The frequency of long-term natural disasters significantly affects the people’s general trust, regardless of whether the respondent lives in the South or in the North; the higher the frequency of natural disasters, the higher the general trust in the region, which is consistent with the results above. Interestingly, the regression of the southern subsample shows that floods have a significant impact on the general trust of the region, while the impact of drought is not significant. The regression of the northern subsample shows just the opposite. A possible explanation is that flood, the disaster that particularly destroys rice production, occurs more frequently in the South, where rice is the dominant crop; therefore, floods have a more significant impact on trust in the South. On the other hand, the main food crop in northern China is wheat, a crop that is fairly fragile to droughts. Unfortunately, droughts occur frequently in northern China, so they generally have greater impacts on trust in the northern areas.

## 5. Conclusions

Social trust is regarded as one of the most important factors for economic growth. Although previous studies have investigated social trust sources from various perspectives, most of them focus on the short-run perspective. This paper measure how long-term natural disasters such as flood and drought affect people’s social trust. We measure long-term natural disaster frequency with flood and drought records from 1470 to 2000. Using social trust data embraced from China Family Panel Studies (CFPS) in 2012, we confirm that long-term natural disasters have a subtle impact on people’s social trust. Indeed, long-term natural disasters and generalized trust show a significant relationship. Moreover, this paper study people’s trust in some specific groups of people. People are more likely to trust neighbors and doctors in regions where long-term natural disasters are more frequent. We further discuss the regional difference in the impact of long-term natural disaster frequency, and we find that flood significantly influences social trust in southern China and drought significantly influences social trust in northern China. As natural disaster conditions vary from country to country, the impact of long-term natural disasters in other countries and regions may differ from China. But, our methodology could be implemented in further research.

History offers variations in natural environment and economic institutions that allow researchers to identify the sources of key parameters of development. Given the context of long-term natural disasters in China, we show that natural disasters in the past has a subsequent impact on current people’s social trust. From a long-term historical perspective, natural disasters not only have a temporal influence but also force people to enhance boundaries and improve social trust permanently. The contribution of this paper is to provide a new perspective on the reasons for the formation of regional social trust from a long-term historical perspective. Human psychology and behavior are the results of external environmental influences, and this influence is often long-term and historical.

## Figures and Tables

**Table 1:** Descriptive statistics of variables.

Variable	Observartions	Mean	Std. Dev	Min	Max
General trust	31,314	0.54	0.50	0	1
Trust in specific groups	29,885	31.68	7.91	0	60
Trust in parents	31,160	9.08	1.70	0	10
Trust in neighbours	31,299	6.37	2.22	0	10
Trust in American	30,160	2.52	2.50	0	10
Trust in strangers	31,173	2.18	2.14	0	10
Trust in cadres	31,174	4.89	2.47	0	10
Trust in doctors	31,273	6.62	2.26	0	10
Natural disaster frequency	34,856	0.59	0.18	0.17	0.89
Flood frequency	34,856	0.29	0.11	0.08	0.45
Drought frequency	34,856	0.22	0.06	0.10	0.50
Gender (1 = male, 0 = female)	34,518	0.50	0.50	0	1
Age	34,851	44.23	16.87	16	99
Marriage (1 = yes, 0 = no)	34,850	0.85	0.36	0	1
Education	34,815	6.70	4.90	0	22
Hukou (1 = urban, 0 = rural)	34,630	0.43	0.49	0	1
Religious beliefs (1 = yes, 0 = no)	31,458	0.11	0.31	0	1
Household income(ln)	33,946	8.95	1.18	−1.61	14.23
Ethnic minorities ratio	34,856	0.17	2.08	−4.61	4.52
Per gdp(ln)	34,856	9.99	1.00	8.05	12.67

**Table 2:** The impact of long-term natural disaster frequency on generalized trust.

Variables	Generalized Trust					
	R1	R2	R3	R4	R5	R6
Natural disaster frequency	1.15 ***	0.93 ***				
	(0.40)	(0.36)				
Flood frequency			1.80 **	1.63 **		
			(0.86)	(0.75)		
Drought frequency					2.18 ***	1.54 **
					(0.80)	(0.72)
Gender (1 = male, 0 = female)		0.05 **		0.05 *		0.05 **
		(0.02)		(0.02)		(0.02)
Age		0.01 ***		0.01 ***		0.01 ***
		(0.00)		(0.00)		(0.00)
Marriage (1 = yes, 0 = no)		−0.36 ***		−0.36 ***		−0.36 ***
		(0.05)		(0.05)		(0.05)
Education		0.07 ***		0.07 ***		0.07 ***
		(0.00)		(0.00)		(0.00)
Hukou (1 = urban, 0 = rural)		0.06		0.06		0.06
		(0.04)		(0.04)		(0.04)
Religion (1 = yes, 0 = no)		−0.06		−0.06		−0.06
		(0.04)		(0.05)		(0.05)
Household income(ln)		0.02		0.02		0.02
		(0.01)		(0.01)		(0.01)
Ethnic minoritities ratio		−0.02		−0.02		−0.02
		(0.02)		(0.02)		(0.02)
Per gdp(ln)		0.02		0.03		0.02
		(0.04)		(0.04)		(0.05)
Province fixed-effect	Yes	Yes	Yes	Yes	Yes	Yes
Constant	−0.39	−1.56 ***	−0.12	−1.47 ***	−0.16	−1.37 ***
	(0.28)	(0.55)	(0.26)	(0.57)	(0.22)	(0.53)
Observations	31,314	30,013	31,314	30,013	31,314	30,013
Pseudo R square	0.03	0.03	0.03	0.03	0.03	0.03

Note: Robust standard errors are in parentheses. *** Significant at 1 percent level. ** Significant at 5 percent level. * Significant at 10 percent level.

**Table 3:** The impact of long-term natural disaster frequency on trust in specific groups.

Variables	Trust in Specific Groups					
	R1	R2	R3	R4	R5	R6
Natural disaster frequency	2.45 *	2.52 *				
	(1.25)	(1.30)				
Flood frequency			1.29	1.01		
			(3.28)	(3.32)		
Drought frequency					7.02 **	7.23 *
					(3.35)	(3.68)
Gender (1 = male, 0 = female)		0.39 ***		0.39 ***		0.39 ***
		(0.09)		(0.09)		(0.09)
Age		0.02 ***		0.02 ***		0.02 ***
		0.00		0.00		0.00
Marriage (1 = yes, 0 = no)		−2.17 ***		−2.16 ***		−2.17 ***
		(0.19)		(0.19)		(0.19)
Education		0.13 ***		0.13 ***		0.13 ***
		(0.02)		(0.02)		(0.02)
Hukou (1 = urban, 0 = rural)		−0.20 **		−0.20 **		−0.21 **
		(0.09)		(0.09)		(0.09)
Religion (1 = yes, 0 = no)		−0.02		−0.02		−0.02
		(0.02)		(0.02)		(0.02)
Household income(ln)		0.04		0.04		0.05
		(0.06)		(0.06)		(0.06)
Ethnic minorities ratio		0.00		−0.02		0.01
		(0.08)		(0.08)		(0.08)
Per gdp(ln)		−0.23		−0.19		−0.25
		(0.19)		(0.19)		(0.19)
Province fixed-effect	Yes	Yes	Yes	Yes	Yes	Yes
Constant	31.81 ***	33.06 ***	33.08 ***	34.85 ***	31.84 ***	34.13 ***
	(0.89)	(2.47)	(1.20)	(2.59)	(0.81)	(2.26)
Observations	29,900	29,000	29,900	29,000	29,900	29,000
Adjusted R square	0.01	0.02	0.01	0.02	0.01	0.02

Note: Robust standard errors are in parentheses. *** Significant at 1 percent level. ** Significant at 5 percent level. * Significant at 10 percent level.

**Table 4:** The impact of long-term natural disaster frequency on trust in different groups.

Variables	Trust in Specific Groups					
	Parents	Neighbors	Americans	Strangers	Cadres	Doctors
Natural disaster frequency	0.2	0.55 ***	0.01	−0.25	0.17	0.57 ***
	(0.26)	(0.18)	(0.26)	(0.23)	(0.21)	(0.18)
Gender(1 = male, 0 = female)	0.02	0.10 ***	−0.03 ***	0.15 ***	−0.03 **	−0.02
	(0.01)	(0.01)	(0.01)	(0.01)	(0.01)	(0.01)
Age	−0.01 ***	0.00 ***	0.00	0.00 ***	0.01 ***	0.00
	(0.00)	(0.00)	(0.00)	(0.00)	(0.00)	(0.00)
Marriage(1 = yes, 0 = no)	0.07 **	−0.11 ***	−0.34 ***	−0.26 ***	−0.26 ***	−0.01
	(0.03)	(0.02)	(0.03)	(0.03)	(0.02)	(0.02)
Education	0.02 ***	0.01 ***	0.02 ***	0.02 ***	−0.01 ***	−0.00 **
	(0.00)	(0.00)	(0.00)	(0.00)	(0.00)	(0.00)
Hukou(1 = urban, 0 = rural)	0.00	−0.01	−0.01	−0.01	−0.03 ***	−0.03 ***
	(0.01)	(0.01)	(0.01)	(0.01)	(0.01)	(0.01)
Religion(1 = yes, 0 = no)	0.00	0.00	0.00	0.00	0.00	0.00
	0.00	0.00	0.00	0.00	0.00	0.00
Household income(ln)	0.01	0.00	0.03 ***	0.01	−0.02 **	−0.01
	(0.01)	(0.01)	(0.01)	(0.01)	(0.01)	(0.01)
Ethnic minorities ratio	−0.01	−0.02 **	0.01	0.00	0.02	0.00
	(0.01)	(0.01)	(0.01)	(0.01)	(0.01)	(0.01)
Per gdp(ln)	0.07 **	−0.03	0.04	0.02	−0.09 ***	−0.06 **
	(0.03)	(0.02)	(0.03)	(0.03)	(0.02)	(0.02)
Province fixed-effect	Yes	Yes	Yes	Yes	Yes	Yes
Observations	30,200	30,300	29,200	30,200	30,200	30,300
AIC	69,552.46	122,657.20	111,856.31	111,327.66	129,383.62	125,778.28
Pseudo R square	0.03	0.01	0.02	0.01	0.01	0.00

Note: Robust standard errors are in parentheses. *** Significant at 1 percent level. ** Significant at 5 percent level.

**Table 5:** The impact of long-term natural disaster frequency on generalized trust: South and North.

Variables	Generalized Trust					
	South			North		
	R1	R2	R3	R4	R5	R6
Natural disaster frequency	2.68 *			0.57 *		
	(1.56)			(0.31)		
Flood frequency		4.21 ***			0.51	
		(1.61)			(0.73)	
Drought frequency			2.91			1.30 *
			(5.63)			(0.66)
Gender (1 = male, 0 = female)	0.03	0.03	0.03	0.06 *	0.05 *	0.06 *
	(0.04)	(0.04)	(0.04)	(0.03)	(0.03)	(0.03)
Age	0.01 ***	0.01 ***	0.01 ***	0.01 ***	0.01 ***	0.01 ***
	(0.00)	(0.00)	(0.00)	(0.00)	(0.00)	(0.00)
Marriage (1 = yes, 0 = no)	−0.43 ***	−0.42 ***	−0.42 ***	−0.31 ***	−0.31 ***	−0.31 ***
	(0.07)	(0.07)	(0.07)	(0.07)	(0.07)	(0.07)
Education	0.06 ***	0.07 ***	0.06 ***	0.07 ***	0.07 ***	0.07 ***
	(0.01)	(0.01)	(0.01)	(0.00)	(0.00)	(0.00)
Hukou (1 = urban, 0 = rural)	−0.04	−0.04	−0.03	0.12 **	0.13 **	0.12 **
	(0.07)	(0.07)	(0.07)	(0.05)	(0.05)	(0.05)
Religion (1 = yes, 0 = no)	0.03	0.03	0.02	−0.13 **	−0.13 **	−0.13 **
	(0.08)	(0.08)	(0.08)	(0.05)	(0.05)	(0.05)
Household income(ln)	0.04 **	0.04 **	0.04 **	0.01	0.01	0.01
	(0.02)	(0.02)	(0.02)	(0.02)	(0.02)	(0.02)
Ethnic minorities ratio	0.03	0.05	0.00	−0.03 *	−0.04 **	−0.03 *
	(0.04)	(0.04)	(0.04)	(0.02)	(0.02)	(0.02)
Per gdp(ln)	−0.01	0.00	0.04	−0.03	−0.02	−0.03
	(0.09)	(0.08)	(0.09)	(0.06)	(0.06)	(0.06)
Province fixed-effect	Yes	Yes	Yes	Yes	Yes	Yes
Constant	−1.40 **	−1.33 **	−1.49 **	−0.62	−0.40	−0.53
	(0.60)	(0.60)	(0.71)	(0.69)	(0.78)	(0.65)
Observations	13,027	13,027	13,027	16,986	16,986	16,986
Pseudo R square	0.0238	0.0238	0.0238	0.0238	0.0238	0.0238

Note: Robust standard errors are in parentheses. *** Significant at 1 percent level. ** Significant at 5 percent level. * Significant at 10 percent level.

## Data Availability

The datasets used and analyzed in the current study are available from the corresponding author on reasonable request.
